# Recent Advances in Studying the Regulation of Fruit Ripening in Tomato Using Genetic Engineering Approaches

**DOI:** 10.3390/ijms25020760

**Published:** 2024-01-07

**Authors:** Denis Baranov, Vadim Timerbaev

**Affiliations:** 1 Laboratory of Expression Systems and Plant Genome Modification, Branch of Shemyakin-Ovchinnikov Institute of Bioorganic Chemistry, Russian Academy of Science, 142290 Pushchino, Russia; d.y.baranov@yandex.ru; 2 Laboratory of Plant Genetic Engineering, All-Russia Research Institute of Agricultural Biotechnology, 127550 Moscow, Russia

**Keywords:** *Solanum lycopersicum* L., CRISPR/Cas9, RNA interference, silencing, overexpression, transcription factors

## Abstract

Tomato (*Solanum lycopersicum* L.) is one of the most commercially essential vegetable crops cultivated worldwide. In addition to the nutritional value, tomato is an excellent model for studying climacteric fruits’ ripening processes. Despite this, the available natural pool of genes that allows expanding phenotypic diversity is limited, and the difficulties of crossing using classical selection methods when stacking traits increase proportionally with each additional feature. Modern methods of the genetic engineering of tomatoes have extensive potential applications, such as enhancing the expression of existing gene(s), integrating artificial and heterologous gene(s), pointing changes in target gene sequences while keeping allelic combinations characteristic of successful commercial varieties, and many others. However, it is necessary to understand the fundamental principles of the gene molecular regulation involved in tomato fruit ripening for its successful use in creating new varieties. Although the candidate genes mediate ripening have been identified, a complete picture of their relationship has yet to be formed. This review summarizes the latest (2017–2023) achievements related to studying the ripening processes of tomato fruits. This work attempts to systematize the results of various research articles and display the interaction pattern of genes regulating the process of tomato fruit ripening.

## 1. Introduction

The fruits of angiosperms are included in the staple diet of humans and livestock. The transition of plants from the vegetative growth phase to the reproductive stage is the main switch in their life cycle. Ripening manifests in bright pigmentation, increased aroma and taste, and softening of the pulp, making the fruits attractive to animals, which act as seed dispersal vectors. It is initiated and regulated by the combined action of various genetic factors in response to endo- and exogenous stimuli. Before the onset of ripening, these physiological changes are suppressed. However, once the fruit enters the ripening phase, it occurs highly synchronized with dramatic alterations in gene expression patterns. Understanding the molecular basses and interrelationships of the regulatory signaling pathway components controlling ripening is biologically interesting but also crucially important for commercial use requiring the high nutritional quality and prolonged storage life of fruits.

**Figure 1:**
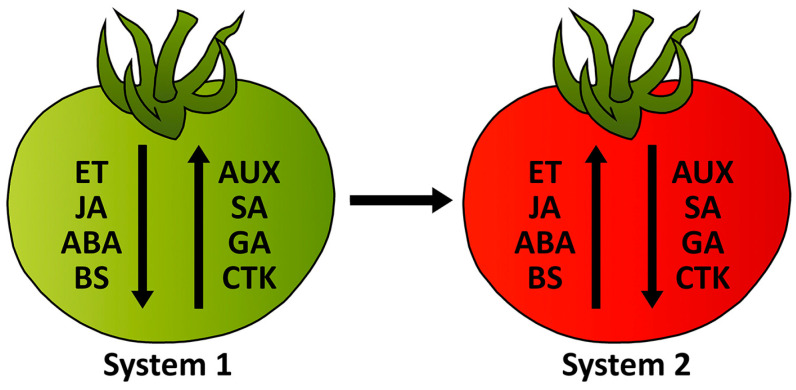
Hormonal changes during transition from ripening system 1 to system 2. Vertical arrows indicate increase or decrease in hormone concentration.

As a commercially important crop (Figure S1), tomato (*Solanum lycopersicum* L.) is grown for both fresh consumption and for in processed forms. It is grown mainly in Asia (Figure S2), while China is the largest tomato fruit producer (Figure S3). Tomatoes’ self-compatibility and short life cycle (90–120 days) enable growers to cultivate them for profit [1]. The development and ripening of tomato fruits depend on two ethylene biosynthetic systems, System I, and System II (Figure 1). Immature fruits and other plant organs continually produce low amounts of ethylene, which System I regulates. As a climacteric fruit, tomato is characterized by an instant increase in ethylene synthesis upon initiation of fruit ripening, which System II mediates [2,3].

Ethylene affects the transcription and translation of many ripening-related genes [4,5] and is controlled by transcription factors [6]. Abruption of ethylene synthesis, perception, or regulation prevents normal fruit ripening [7]. Nevertheless, it has been proposed that both ethylene-dependent and ethylene-independent gene regulation pathways coexist to coordinate the process of ripening in fruit, even though ethylene is the predominant trigger for ripening in climacteric fruit [8].

In addition to ethylene, there are many signaling cascades that regulate the former expression and facilitate the accumulation of metabolites in tomato fruits. Tomato fruit is rich in primary metabolites like sucrose [9,10,11,12], hexoses [13,14,15], organic acids [16,17,18,19,20,21,22,23], and amino acids [24]. Tomato fruit also contains various secondary metabolites, including pigments, mostly lycopene [25,26,27] and beta-carotene [28,29], and antioxidants, namely flavonoids [30,31,32,33,34], and ascorbic acid [35,36,37,38].

Besides the accumulation of natural metabolites, tomato fruit can produce a foreign one. For instance, tomatoes do not synthesize tyrosine-derived compounds, betalains, used as food coloring or as antioxidants. The authors of [39] transferred a betanin biosynthesis gene cassette into a tomato, which showed high expression efficiency. Glycine betaine is also not synthesized in tomato. The transfer of betaine aldehyde dehydrogenase and choline oxidase genes into tomato induces the formation of enlarged flowers and fruits in transgenes [40]. Mogrosides are used as sugar substitutes and characterized by their high sweetness, low calorie content, and non-toxicity; recently, a expression cassette with six mogroside III synthase genes was successfully transferred into tomato [41]. In addition, transgenic tomatoes that produce and accumulate vaccines in fruits are promising. Although it faces certain difficulties, such as the low concentration of the produced protein in cells or differences in post-translational modification of proteins, research is nevertheless being carried out [42,43,44,45].

In addition to the nutritional importance of tomatoes, it is a convenient object for studying the mechanisms of climacteric fruit ripening regulation since their functions are often conserved. This is due to tomato’s simple diploid genetics, small genome size [46,47], ease of transient and stable transformation [48,49,50,51,52], and pronounced ripening phenotypes. Also, many well-characterized tomato mutants are altered in fruit development and ripening, and for most of them, the underlying genes have been identified. [53,54,55,56,57,58,59].

Modern widespread varieties of tomatoes were obtained through domestication and subsequent selection. The selection of seemingly desirable traits carried out without an understanding of the nature of gene relationships has contributed to the reduction in genetic diversity in tomatoes. Consumer preferences and cultivation convenience have also contributed to this. On the other hand, the introgression of alleles from wild tomato or tomato relatives into a cultivar helps create a hybrid genome with the allele of interest but also introduces undesirable genetic backgrounds in the form of linked genes from the donor. With the help of backcrossing, breeders can level out the manifestation of unwanted traits, but this is time-consuming and not consistently effective.

Genetic engineering methods provide significant potential for studying the genetic factors regulating fruit ripening. Thus, targeted genome editing technology using the CRISPR/Cas9 system allows researchers to create allele knockout and make precise changes to the gene sequence. This, in turn, helps us to study genes’ functions, relationships, and regulation. Despite the 10-year history of using this powerful technology, the number of publications using it, in which the tomato is the object, is growing steadily yearly (Figure S4). Thus, its research potential still needs to be exhausted.

**Figure 2:**
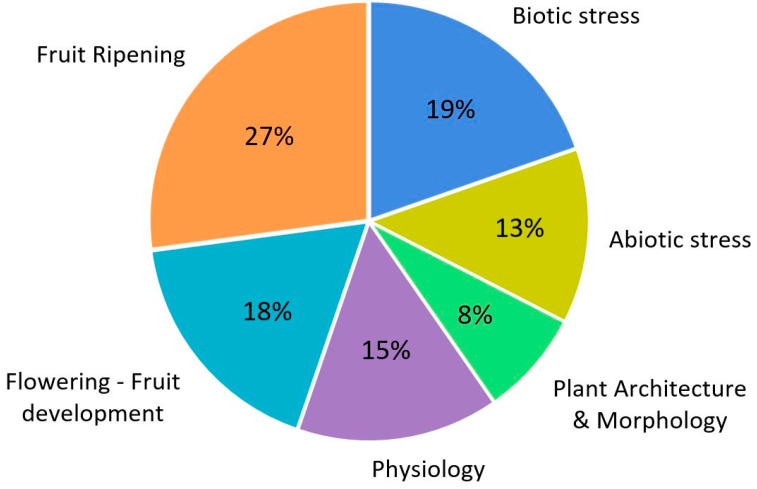
Topics of studied tomato genes (2017–2023) by CRISPR/Cas9.

An analysis of publications where the CRISPR/Cas9 system has been used in tomatoes over the past six years has made it possible to identify the topics of most interest to the scientific community (Figure 2). It turned out that more than a quarter of the total number of works are devoted to the study of genes involved in the processes of fruit ripening (27%). Many of these genes encode transcription factors and transcriptional coregulators, microRNAs, or proteins involved in the epigenetic control of gene expression. In many cases, these regulators’ molecular mechanisms of action have yet to be studied, which is the reason for the growing interest in research in this area. Also, a considerable proportion of publications are devoted to the study of the regulation of the processes of flowering and fruit development (18%). The consistently current topic of stress (abiotic and biotic) occupies a third of the total number of publications. The remaining publications cover fields devoted to other physiological processes (15%) and plant architecture and morphology (8%).

**Figure 3:**
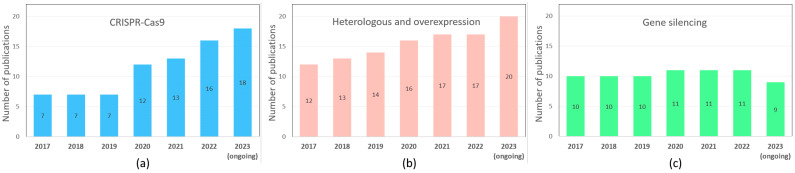
The number of publications devoted to exploring the processes of flowering and ripening in tomatoes: using CRISPR/Cas9 technology (**a**), using over- and heterologous expression approach (**b**), and using gene silencing technologies (**c**).

Among the most prevalent genetic engineering methods used to study the regulation of ripening processes, the use of the CRISPR/Cas9 system is expected to increase (Figure 3a). At the same time, approaches that have already become classical, such as RNA interference gene silencing, and gene over- and heterologous expression, have not lost their relevance—the number of publications using them has remained consistently high over the past six years (Figure 3b,c). Interestingly, there are a growing number of studies using gene overexpression to study ripening. All this suggests that, despite the large amount of accumulated data, the regulation of the ripening process still needs to be fully understood.

This review highlights new advances in understanding aspects regulating tomato fruit ripening using CRISPR/Cas9 targeted gene editing, RNA interference, and gene overexpression. Here, we highlight all components that mediate ripening, namely regulatory pathways, transcription factors, epigenetic modifications, and abiotic factors. In the end, based on collected data, we propose a molecular interaction network model of ripening signaling pathways in tomato.

## 2. Transcription Factors Regulating Ripening

MADS-box genes are among the most widely represented and diverse transcription factors; consequently, they mediate various biological processes. Among them, regulating fruit ripening is one of the most prominent roles of MADS-box (*MCM1*, *AGAMOUS*, *DEFICIENS*, and *SRF*) genes. The transcription factor RIN (RIPENING INHIBITOR) has long been considered a major ripening regulator. *RIN* encodes a SEPALATA class MADS-box transcription factor. MADS-box family transcription factors typically function as multimers, and the MADS-box proteins TAGL1 (TAG-like) and two FRUITFULL (FUL) homologs (TDR4/FUL1 (tapetum degeneration retardation) and MBP7/FUL2 (MADS-box protein)), are coregulators with RIN and ripening regulators with overlapping functions [60]. Silencing of *TAGL1* resulted in decreased levels of amino acids in fruit: aspartic acid, L-tyrosine, L-glutamine, L-phenylalanine, L-valine, L-leucine, isoleucine, and 5-caffeoylquinic acid [61]. TAGL1 was also found to regulate the synthesis of the glycoalkaloid α-tomatine negatively. As discussed in [62], TDR4/FUL1 and MBP7/FUL2 do not regulate ethylene biosynthesis but influence fruit ripening in an ethylene-independent manner. RIN often binds to demethylated sites in the promoter regions of ripening-related genes. RIN is induced early in ripening and stimulates ethylene-dependent and ethylene-independent pathways that promote ripening. Mediators in this process are response factors to ethylene (ERF, ethylene-responsive factor) and auxin (ARF, auxin-response factor). ERF and ARF control their respective hormonal signaling pathways, regulating gene expression and hormonal signaling.

Several recent studies have clarified the function of RIN. Thus, [63] found that although RIN function is required for full ripening, RIN is not required for the initial ripening induction. The authors suggest that RIN acts redundantly (i.e., there are RIN homologs) or RIN-independent ripening induction occurs due to other transcription factors. In the second case, the authors concluded that an RIN-independent activator can induce the transcription of ripening-related genes even in RIN-deficient plants. Still, a mutant (defective) RIN protein can inhibit its activity. The chimeric transcription factor RIN-MC exhibits a negative role in ripening, promoting the mutant *rin* phenotype [64]. Other authors think that low ethylene concentrations initiate the ripening of mature green fruits, activate RIN expression, and lead to other changes, including a transition to a burst of autocatalytic ethylene synthesis [65]. Combined with the ethylene biosynthesis gene *ACS2* (1-aminocyclopropane-1-carboxylate synthase), RIN has been shown to regulate the heat shock genes *HSP17.7* [66] negatively. Therefore, RIN, ethylene, and other factors are necessary to complete the complete fruit ripening program. RIN is not only an activator of ripening but also a repressor of excessive softening [67]. It was found that when controlling the fruit ripening process, RIN binds to six lncRNAs [68].

RIN is reported to directly activate the expression of a novel gene, *E6-2*, involved in tomato fruit ripening [69]. The silencing of *E6-2* leads to a delay in the fruit-ripening suppression of *CNR* (colorless non-ripening), *PG* (polygalacturonase), and *ERF4* (ethylene-responsive factor), a decrease in the accumulation of carotenoids and lycopene (due to the suppression of *PSY1*, *PDS* and *ZDS* (phytoene synthase, phytoene desaturase, and zeta-carotene desaturase, respectively)), and ethylene (decreased expression of the biosynthetic genes *ACS2*, *ACO1* (1-aminocyclopropane-1-carboxylic acid oxidase), *ACO3* and ethylene-sensitive *E4*, *E8*), and an increase in the content of pectin, cellulose, starch and soluble sugar (suppression of cell wall metabolism genes *TBG4* (tomato beta-galactosidase), *PL* (pectate lyase), *EXP1* (expansin), and *XTH5* (xyloglucan endotransglucosylase/hydrolase)). The broad phenotypic pattern of *RIN* silencing is an attractive marker for testing molecular editing tools [70,71,72].

Genes with the NAC domain (NAM, ATAF1/2, and CUC2 (apical meristem, ARABIDOPSIS TRANSCRIPTION ACTIVATOR FACTOR, and cup-shaped cotyledon, respectively)) are considered to be other transcription factors that regulate tomato fruit ripening. It has been shown [73] that the inhibition of *NOR-like1* reduces ethylene production, delayed softening and loss of chlorophyll, and reduced lycopene accumulation. Activation of ethylene synthesis genes by *NOR* (non-ripening) and *NOR-like* genes is discussed further in [74]. The knockout of *NAC-NOR* suppressed fruit ripening (inhibition of ethylene synthesis, reduction in carotenoid accumulation, and fruit softening), and the opposite effect was observed with its overexpression [7]. The replacement of thymine with adenine in the *ALC* gene (alcobaca, NOR mutation) using homologous recombination contributed to an increase in the shelf life of tomato fruits [75]. Expression of peach *NAC1* in tomatoes has been shown to enhance ripening in a delayed ripening (NOR) mutant and restore the synthesis of volatile esters [76]. Overexpression of *NAC6* resulted in increased levels of endogenous abscisic acid, which affected the transcription of ripening genes [77]. Transfer of the kumquat *NAC22* gene to tomato increased the expression of most carotenoid biosynthesis genes, accelerated the transformation of plastids into chromoplasts, and promoted color changes [78]. NAM1 (no apical meristem), another factor with an NAC domain responsible for the regulation of ethylene biosynthesis, also controls tomato ripening, as confirmed by delayed ripening in CRISPR/Cas9 mutants and accelerated ripening for lines overexpressing *NAM1* [79]. Repression of *NAM* gene domains is carried out by miR164a [80,81,82]. In addition, the *HWS* (HAWAIIAN SKIRT) gene, encoding an F-box protein, regulates the number of floral organs by modulating the transcription levels of the *miR164*, *CUC1* and *CUC2* (cup-shaped cotyledon) genes. HWS is also involved in petals’ cell proliferation and mitotic growth [52].

It was previously shown that a representative of genes with the NAC domain *NAP2* (*Arabidopsis* NAC domain-containing protein) activates the aging gene *SAG113* (senescence-associated gene), protein phosphatase), chlorophyll degradation genes *SGR1* (stay-green), *PAO* (polyamine oxidase), and *NAP2*, and also directly controls the expression of genes essential for the biosynthesis of abscisic acid *NCED1* (9-cis-epoxycarotenoid dioxygenase), *ABCG40* (*Arabidopsis thaliana* ATP-binding cassette), and *CYP707A2* [83]. These interactions suggest the influence of NAP2 on leaf senescence and yield in tomato. Another new gene, named *HEBE* by the authors in honor of the Greek goddess of youth, has similar functions [84].

Numerous studies have shown that the *CNR* gene is the most important regulator of tomato fruit ripening. However, recent research [85] has called this assumption into question. *CNR* knockout lines exhibited only a ripening-arrested phenotype, while *NOR* knockout (non-ripening) lines exhibited a partial non-ripening phenotype similar to RIN mutants. Both knockouts differed from the strong, non-ripening phenotypes of their natural mutants. It became apparent that the expression of characteristic ripening genes, such as *ACS2*, *ACO1*, *PSY*, *PG*, and *EXP*, is not entirely suppressed in CRISPR/Cas9 lines compared to natural mutants. As the authors concluded, differences in the expression of the genes in question are explained by different degrees of methylation, and they also concluded that the regulatory network of transcription factor genes is redundant. Regulation of NOR may also be associated with something else: sulfoxidation of the NOR transcription factor with the help of MSR (methionine sulfoxide reductase) proteins modulates the ripening process by reducing the DNA-binding ability of NOR [86].

It is known that the fruits of the tomato epimutant *cnr* fail to ripen and remain colorless. The *SPL* (SPOROCYTELESS) gene family consists of a group of genes encoding SBP (SQUAMOSA promoter binding proteins)-box transcription factors, and their protein products bind to the promoter of the floral meristem identity gene *SQUAMOSA*. Evidence shows that SPL-CNR interacts with SnRK1 (SNF1-related protein kinase) [87]. The suppression of *SnRK1* by virus-induced gene silencing (VIGS) inhibits fruit ripening and leads to decreased expression of a wide range of ripening-related genes. This suggests that *SnRK1* transcription and subsequent post-translational SPL-CNR-SnRK1 interaction are biologically crucial for tomato fruit ripening. The authors suggest that the involvement of SnRK1 in fruit ripening may be due to the physical interaction of proteins between the *SnRK1* gene product and SPL-CNR and subsequent phosphorylation of SPL-CNR due to the kinase activity of SnRK1 [87].

The role of some MBP transcription factors in the ripening process has recently been studied. Thus, suppression of the MBP8 factor shortened the fruit ripening time, suggesting an increase in the activity of ethylene synthesis genes [88]. Meanwhile, carotenoids accumulated to higher levels, and the expression of *PSY1*, *PDS*, and *ZDS* was enhanced in *MBP8* RNAi-silenced fruits. The activity of cell wall genes also changed, manifested in the softening of fruits. Silencing *MBP15* in [89] delayed tomato ripening, and gibberellin, carotenoid, and ethylene biosynthesis genes were repressed. MBP15 was found to interact with RIN [89].

GRAS (gibberellic acid insensitive (GAI), repressor of GAI (RGA), and scarecrow (SCR)) proteins are plant-specific transcription factors that play critical roles in plant development and stress response. It turned out that they also take part in regulating fruit ripening. For example, silencing *GRAS2* reduces tomato fruit weight, which has been attributed to insufficient levels of gibberellic acid during initial ovary development [90]. Overexpression of *GRAS4* accelerated fruit ripening (due to the activation of expression in the promoter region of ethylene biosynthesis genes and repression of the negative regulator of ripening MADS1). It increased the total content of carotenoids [91]. GRAS24, in addition to flowering and ripening, is responsible for a variety of other agronomic traits, including plant height, leaf architecture, number of lateral branches, root length, and the observed pleiotropic effects in plants overexpressing *GRAS24* are due to impaired modulation of gibberellin and auxin signaling [92].

Transcription factors of the WRKY superfamily exhibit upregulation during fruit ripening. WRKY32 binds to W-box and similar motifs in the regulatory region of the *YFT1* (yellow-fruited tomato) promoter and induces its expression [93]. *YFT1* encodes the EIN2 protein, a major ethylene signal transduction component. Suppression of ethylene production resulted in delayed chromoplast development, decreased carotenoid accumulation, and a yellow fruit phenotype. Twelve *WRKY* genes were also shown to be ethylene-responsive (ER), eight of which activated the promoters of color change-associated genes *PPH* (pheophytinase), *PAO* (polyamine oxidases), *PSY1*, and *PDS* [94]. In addition, protein interactions were found between WRKY17 and RIN/ERF2b/ERF7, WRKY33 and ERF7, WRKY54 and ERF2b, WRKY16 and WRKY1, which only confirms the complexity of the networks of ripening regulators [94].

## 3. Epigenetic Modifications as Regulators of Ripening

Heritable variations in gene expression that take place without affecting the underlying DNA sequence are referred to as epigenetics. They are transmitted via cell division and DNA replication, establishing and preserving gene expression patterns unique to particular cell types [95,96,97,98].

DNA methylation has a critical role in a wide range of cellular functions. For example, a decrease in DNA methylation levels can be observed during fruit ripening, which is explained by DNA demethylase (DML) activation. In *DML2* loss-of-function mutants generated by targeted editing, increased DNA methylation was found not only in genes induced during ripening but also in genes repressed during ripening [99]. However, a recent study found that the highly mobile protein positively regulates *DML2* expression of 3-hydroxy-3-methylglutaryl-coenzyme A reductase (*HMGA*) [100]. In [101], the expression of the mammalian demethylase TET3c (ten-eleven translocation) in tomatoes resulted in the activation of expression of the previously undescribed gene *CEN1.1*. The activation intensity of *CEN1.1* expression correlated with increased hypomethylation in its promoter, suggesting that *CEN1.1* expression is associated with the DNA methylation of CHH promoter sites (H = A/C/T). Phenotypically, *CEN1.1* emerged as a repressor of flowering in tomatoes, leading to the development of leaves on inflorescences. Paradoxically, this led to an increase in the number of fruits but to a longer time for their ripening. Thus, this study provides an exciting approach to identifying methylation-associated genes.

In plants, cytosine methylation plays a crucial role in suppressing the movement of transposable elements. Methylation is maintained by DNA methyltransferases MET1, and CMT3 (chromomethylase), as well as additional proteins (for example, DDM1 (decreased DNA methylation)) involved in maintaining a heterochromatic structure. The methylase encoded by *MET1* is a key DNA methylase responsible for maintaining CG methylation in plants. Loss-of-function mutants of the *MET1* gene had pleiotropic developmental phenotypes manifested as small curled leaves, defective flowers, and parthenocarpic fruits [102]. Also, the knockout of *MET1* resulted in changes in the expression profiles of *RIN* target genes, such as *ACC2* (acetyl-CoA carboxylase 2). In another study, suppression of *MET1* by VIGS in a hypermethylated epimutant *CNR* promoted vivipary development [103]. The authors explain this by a decrease in the concentration of abscisic acid and *NCED* transcripts involved in its biosynthesis. The *NCED* silencing had similar consequences. Methyltransferase DRM7 (domains rearranged methyltransferase) has been shown to influence chloroplast development by modulating starch accumulation and chlorophyll synthesis. It has an epi-effect on leaf senescence, affecting tomatoes’ vegetative growth [104]. The transient expression of arginine methyltransferase *PRMT1.5* in tomatoes inhibited the accumulation of carotenoids and anthocyanins [105].

Short interfering RNAs (siRNAs) also mediate DNA methylation through RNA-directed DNA methylation (RdDM). It has been established that heterochromatic mobile elements in plants with *DDM1* dysfunction are deprived of mCG and mCHG, which generally keep them inactive [106]. Methylation of CHH sites increased for some heterochromatic transposons and, conversely, decreased for those localized in euchromatin. Knockout of *CMT4* chromomethylase caused severe morphological changes in tomato plants, accompanied by defects in leaves, pollen, and seeds [107].

Another mode of epigenetic regulation is post-translational modifications of histones. Histone acetylation is known to be associated with gene activation. In contrast, histone methylation can be associated with either activation or repression depending on the lysine residue and the number of methyl groups added. In [108], RNA-seq profiling showed a significant increase in the expression of methylases MET1 and CMT3 and a minor increase in the demethylase DML2 during the fruit set, which is associated with their role in maintaining post-replication DNA methylation during extensive cell division characteristic of early stages of development of the fetus. However, their abundance was significantly lower than that for histone marks H3K9ac and H3K4me3, determined using chromatin immunoprecipitation sequencing. This implies that changes in the transcriptional profile underlying the fruit set are more closely related to histone modifications than methylation. Histone modification is based on the histone methyltransferase genes *SDG27*, *SDG5*, and *SDG16* (set domain group). However, the authors could not create homozygous loss-of-function mutants of these genes, suggesting their exceptional biological importance. Mutants heterozygous for these genes exhibited parthenocarpic fruits. The function of other histone lysine methyltransferases SDG33 and SDG34 was revealed in [109]. They were found to regulate the expression of nitrogen-responsive genes and physiological changes in an organ-specific manner.

It has been demonstrated that histone demethylation leads to activating tomato fruit ripening genes [110]. Here, the *JMJ6* (Jumonji C-terminal domain-containing demethylase) gene was found to encode a histone lysine demethylase that specifically demethylates H3K27. Its overexpression accelerates the ripening of tomato fruits, which is associated with increased expression of the *RIN*, *ACS4*, *ACO1*, *PL*, and *TBG4* genes. As the study [111] showed, *JMJ4* mediates abscisic acid-induced leaf senescence in tomatoes.

By knocking out the *HTA1* genes of histone H2A and subsequent production of double homozygous mutants, changes were identified in the expression patterns of many biological ripening processes, including cell redox homeostasis, mRNA splicing, cell cycle regulation, translation, etc. [112]. Moreover, for three genes of carotenoid biosynthesis, *PSY1*, *PDS*, and *VDE*, expression was high regardless of the fruit ripening stage [112]. Histone deacetylation has been associated with transcriptional repression. Histone deacetylases carry out this process. There is evidence that they can act as both positive and negative ripening regulators. Indeed, RNAi silencing of the *HDT3* (histone deacetylase) gene led to the suppression of genes for ethylene synthesis (*ACS2*, *ACS4*, *ACO1*, and *ACO3*), carotenoids (*PSY1*), cell wall metabolism (*HEX* (acetylhexosaminidase), *MAN* (mannosidase), *TBG4*, *XTH5*, and *XYL* (xylanase)), as well as general genes associated with ripening (*RIN*, *E4*, *E8*, *PG*, *Pti4*, *LOXB* (lipoxygenase)) [113]. In contrast, in [114,115], silencing of the *HDT1* gene led to opposite results for these same transcripts. In this regard, the molecular mechanisms of regulation of these genes remain to be studied.

Histone acetyltransferase GCN5 acetylates histone H3 lysine (H3K14ac) and affects the levels of H3K9ac and H3K27ac. Its suppression leads to the loss of shoot apical dominance and a decrease in the size of the plant apical meristem [116]. It has also been established that GCN5 can increase *WUSCHEL* transcript levels. The expression of *WUSCHEL* can also be regulated by chromatin remodeling factors, such as the histone deacetylase HDA19 [117]. Here, the deacylation mechanism was found to involve the inhibitor gene *IMA* (inhibitor of meristem activity) acting as an adapter protein to form a chromatin remodeling complex together with the zinc finger protein C_2_H_2_ KNU (KNUCKLES) and the transcriptional corepressor TOPLESS.

## 4. Hormonal Control of Ripening

### 4.1. Auxin Regulation

Auxin regulation is involved in all plant processes, including cell elongation and division, the formation of the architecture of roots, leaves, and inflorescences, the development of embryos and fruits, and responses to stress [118,119,120,121]. The primary plant organs of auxin biosynthesis are young leaves and their primordia [122]. From them, YUCCA (YUC)-type flavin-containing monooxygenases catalyze the rate-limiting irreversible reaction: the oxidative decarboxylation of indole-3-pyruvate acid to indole-3-acetic acid (IAA) [123]. Knockout of any of the auxin synthesis genes is associated with lethal phenotypes, so attention is paid to genes providing auxin-mediated inactivation (GH3, GRETCHEN HAGEN), transport (PIN, ABCB (PIN-FORMED, ATP binding cassette subfamily B, respectively)), and signal transduction (ARF (auxin response factor), Aux/IAA).

By conjugating auxins to amino acids for storage or degradation, members of the *GH3* family, encoding acyl acid amidosynthetases, are critical for maintaining auxin homeostasis. In tomatoes, GH3.15 has been shown to regulate lateral root development and response to gravitropism by modulating auxin homeostasis [124], GH3.8 controls plant height [125], GH3.4 negatively regulates mycorrhization [126,127], and GH3.2 affects fruit ripening in the early stages [128].

PINs are one of the facilitators of intercellular auxin transport. VIGS *PIN1* accelerates flower abscission by increasing the accumulation of auxin in the ovule and reducing the auxin content in the abscission zone [129], and its negative regulator is the transcription factor MBP9 [130].

ARFs are plant-specific transcription factors that directly bind to auxin response elements in the promoters of auxin-responsive genes. ARF5 has been shown to regulate fruit set and development [131], ARF10 is involved in the accumulation of chlorophyll and sugar during fruit ripening [132], ARF19 is involved in leaf development [133], several ARFs (ARF6A, ARF8A, ARF8B, and ARF24) interact with the transcriptional repressor IAA9 [134] and regulate leaf shape [135], and ARF10A is essential for the growth of leaf blades and formation of floral organs [136].

### 4.2. Gibberellin Regulation

Gibberellins (GAs) are tetracyclic diterpenoid compounds with a high structural variation, but only a few function as plant hormones in higher plants [137]. GAs are formed primarily from the methylerythritol phosphate pathway [138]. The catalyzes of *trans*-geranylgeranyl diphosphate to *ent*-kaurene occurs in proplastids [139]. This reaction is mediated by *ent*-copalyl diphosphate synthase and *ent*-kaurene synthase [140]. Then, *ent*-kaurene is oxidized to GA_12_ in six steps [141], and catalyzed by *ent*-kaurene oxidase and *ent*-kaurenoic acid oxidase in the endoplasmic reticulum [139]. Finally, GA_12_ is oxidized by 2-oxoglutarate-dependent dioxygenases in the cytosol and the cell nucleus [142,143]. As phytohormones gibberellins regulate various physiological processes of plants, they promote plant growth, participating in stem elongation, the expansion of leaf blades, pollen development, flowering, ripening and seed germination.

DELLA (*GRAS* gene encodes protein containing D-E-L-L-A amino acid sequences) proteins are nuclear-localized negative growth regulators. Gibberellins promote DELLA degradation by assembling the E3 ubiquitin ligase complex, followed by protein degradation. DELLA is encoded by the *PROCERA* gene, and its loss of function in the homozygous state results in dwarfism [144] and parthenocarpy [145]. The degradation of proteins, including DELLA, is controlled by a complex regulatory network involving connections between several signaling pathways [146]. DELLA proteolysis is mediated by the gibberellin-activated receptor GID. Knockout of their coding genes also results in a dwarf phenotype [147]. There is evidence of cross-signaling between the gibberellin and abscisic pathways [148], and the DELLA protein is an activator of abscisic acid transporters (AIT), regulating transpiration through stomatal closure [149]. Tomato PROCERA activity is assumed to be necessary to transition tomatoes to flowering. DELLA protein directly or indirectly promotes the expression of *SFT* (SINGLE FLOWER TRUSS) in leaves, as well as *SBP* and *AP1/MC*, together with microRNAs in the shoot apex [150].

Recently, factors mediating gibberellin-dependent regulation have also received attention. Thus, silencing of the *GRAS15* transcription factor gene led to pleiotropic phenotypes, including reduced plant height, small leaf size with pointed edges, as well as an increased number of nodes, lateral shoots, and petiole length, which is explained by the suppression of gibberellin synthesis genes [151]. The helix–loop–helix transcription factor gene *PRE2* is induced by gibberellin. Its silencing has been shown to cause reductions in fruit size, seed size, pericarp thickness, and placental size [152]. These changes are associated with the decreased expression of xyloglucan endotransglucosylases XTH2 and XTH5. PREs regulate many processes—their overexpression in tomatoes leads to multiple morphological changes, including changes in leaf angle, internode length, leaf curl, and pigment composition [153,154].

Gibberellins antagonize ethylene accumulation during tomato ripening. The delayed metabolic shift mediates GA through the upregulation of auxin signaling [155].

### 4.3. Cytokinin Regulation

Cytokinins promote the development of shoots, provide stress resistance, and delay aging [156,157,158,159]. These isopentenyladenine derivatives are formed mainly in the roots and transported to the aerial parts. The main rate-limiting enzyme for cytokinin synthesis is isopentenyltransferase (IPT) [160]. It has been shown that overexpression of the IPT gene leads to significant phenotypic changes and slower leaf senescence only under the control of a root-specific promoter [161]. IPT4 has been shown to be involved in tomato lycopene biosynthesis [162].

Cytokinin catabolism is carried out by cytokinin oxidases (CKX). The overexpression of CKX2 in tomato fruit decreased cytokinin levels [163]. It is also shown here that endogenous cytokinins regulate the division of pericarp cells, which subsequently determines the size of the fetus.

High levels of cytokinins are often found in the flesh of immature fruits but decrease rapidly at around the time of fruit ripening onset and kept low later. It shows a role in early fruit development, particularly cell division, and in inhibiting ripening [164]. Gibberellins biosynthesis genes are inhibited by DNA hypomethylation during ripening [165].

### 4.4. Ethylene Regulation

Ethylene (ET) is the simplest unsaturated hydrocarbon with the formula C_2_H_2_. It acts as a global regulator of developmental processes and defense in plants. [3,166,167,168]. The ethylene biosynthetic pathway includes three steps [169]: S-adenosylmethionine synthetase (SAMS) modifies methionine to form S-adenosylmethionine (SAM), SAM is converted to 1-aminocyclopropane-1-carboxylic acid (ACC) by synthase (ACS), and in the last step ACC converts ACC oxidase (ACO) with the formation of ethylene.

In studies on tomatoes, ethylene is considered a participant in signaling cascades, including during the ripening process. ET accelerated fruit ripening with the simultaneous repression of auxin signaling [155]. It has been established that its synthesis during ripening is presumably regulated by FER receptor kinases (FERONIA). FERL6 and FERL1 were found to interact physically with the *SAMS* promoter [170]. Expression of *FER* genes in tomatoes showed negative regulation of ethylene accumulation at the initial stages of fruit development and, as a consequence, delayed fruit ripening.

The promoter of the transcription factor *EIN3* gene (ethylene insensitive) has been shown to contain several motifs associated with hormones influencing fruit development and ripening [171]. Overexpression of *EIN3* in tomatoes resulted in the activation of the expression of ethylene biosynthesis genes *ACO1*, *ACS1*, and *SAMS1*, which promoted early fruit ripening. Accordingly, *EIN3* silencing showed the opposite effects. An *EIN3-like* gene causes premature onset of ovule senescence [172].

Ethylene is bound by a family of ETR (ethylene receptor) proteins located in the membrane of the endoplasmic reticulum. ETRs have functional redundancy. ETR3-mediated signaling inhibits pollen tube growth without sufficient ethylene [173]. ETR3 promotes the activation of cell wall remodeling genes and Ca^2+^ transporters—overexpression of *ETR7* results in earlier flowering, short plants, and small fruits [174]. Targeted base substitution in *ETR1/2* causes a delay in ripening and ensures prolonged storage of fruits [175,176].

Ethylene response factors (ERFs) are signaling components involved in ethylene-dependent developmental processes. They can perform both the positive and negative regulation of target genes. Their number is large, as is the specificity of the reactions of tomato genes to ethylene: the regulation of fruit ripening processes [177,178,179,180], control of aging [181], participation in the activation of protective reactions [182,183,184], growth [185,186], accumulation of chlorophyll and formation of chloroplasts [187], and regulation of other signaling pathways [188].

### 4.5. Brassinosteroid Regulation

Brassinosteroids (BS), which include various polyhydroxylated steroidal phytohormones, influence many critical agronomic traits related to growth, photosynthesis, morphology, and yield [189,190,191,192]. The synthesis of BS occurs along three pathways, in which campesterol is the initial substrate [193]. Crosstalk between BS and redox signals suggests a direct involvement of the former in the plant response to stress [194,195]. However, recent studies also reveal a connection between the brassinosteroid and ethylene pathways. Recently, it was demonstrated that overexpression of one of the genes for the brassinosteroid synthesis enzymes DWARF (DWF) in tomatoes promotes fruit softening, lycopene synthesis, and ethylene production, while gene knockout inhibits them [196]. It was concluded that APETALA2a (AP2a) promotes ethylene signaling to regulate BS signaling. Also, tomatoes with overexpression and silencing of the cytochrome P450 monooxygenase *CYP90B3* gene showed a correlation in the content of bioactive BS with the processes of tomato fruit ripening, including softening, the content of soluble sugars and aromatic volatiles [197].

Research into brassinosteroid-dependent pathways is ongoing. The specific receptor for brassinosteroids is BRI1 (BRASSINOSTEROID INSENSITIVE1). Upon binding of BS to its extracellular domain, dimerization of BRI1 and the coreceptor BAK1 (BRI 1-associated receptor kinase 1) occurs. The signal is then transmitted through a phosphorylation cascade involving BSK1 (BR-signaling kinase 1), CDG1 (constitutive differential growth), BSU1 (BRI1 SUPPRESSOR), and BIN2 (BRASSINOSTEROID INSENSITIVE2). Subsequently, BIN2 is inactivated, and two transcription factors, BZR1 (brassinazole resistant) and BES1 (BRI1-extra microsporocytes-suppressor 1), are dephosphorylated by protein phosphatase PP2A. BZR1, BES1, as well as other nuclear factors (for example, BIM1) are regulators of brassinosteroid-dependent genes.

Overexpression of *BRI1* [198] in tomatoes improved carotenoid accumulation by increasing the expression of *DXS* (1-deoxy-D-xylulose 5-phosphate synthase), *GGPS* (geranylgeranyl pyrophosphate synthase), and *PSY1*. In addition, BS induced the expression of genes involved in its ethylene biosynthesis (*ACO1* and *ACS2*). Similar results were achieved by modifying threonine-1050 BRI1, resulting in plants with high levels of BRI1 autophosphorylation [199]. A recent study on BRI1 showed that the receptor also positively regulates a tomato’s tolerance to cold stress [200]. BSs are capable of inducing early flowering. This is supported by the interaction of the suppressor of BIN2 signaling with the early flowering locus *FRIGIDA* [201]. There is evidence that canonical signaling pathways initiated by BRI1 are involved in xylem differentiation and wood formation in tomatoes through activation of the BZR1/2 transcription factors [202]. The BZR1 homolog has been shown to interact with BIM1 to act as a negative regulator of pericarp cell expansion [203]. According to available information, BZR1 is also a trans-activator of the promoter of the *SUN* gene (encodes Sad1/Unc-84 (SUN)-domain proteins), responsible for elongation of tomato fruits, and *BZR1*-knockout tomato phenotypes show redundancy of its homologs [204]. Furthermore, BSs promote tomato bud growth through the direct transcriptional regulation of *BRANCHED1* (*BRC1*) via the signaling component BZR1 [205].

In the study [206], the authors focused on BES1, a key transcription factor in the brassinosteroid signaling pathway. BES1 was found to bind to the promoter of the fruit-softening inhibitor *PMEU1* (pectin methylesterase). Knockdown or knockout of *BES1* in tomatoes resulted in increased shelf life without negatively affecting the appearance and nutritional composition of the fruit.

Altered regulation of BS may influence cell elongation and division, leading to altered fetal morphology. For example, a premature stop codon at the *GLOBE* locus containing a brassinosteroid hydroxylase sequence resulted in a spherical phenotype of tomato fruit, which had a flattened shape in the wild type [207]. Since GLOBE and FW3.2 (KLUH) were found to be members of the same cytochrome P450 family, the authors hypothesized that both may act similarly in regulating fruit size and shape.

During oxidative stress following pesticide application, plants use glutathione to clear excess reactive oxygen species. BS induce pesticide metabolism by activating *GRX* (glutaredoxin) gene expression through transcription factors [208,209].

### 4.6. Abscisic Acid Regulation

Abscisic acid (ABA) is a plant growth regulator, and it regulates seed maturation, seed dormancy, adaptive responses to biotic and abiotic stresses, and abscission of leaves and buds [210]. ABA is produced from the oxidative cleavage of carotenoids [211,212]. This is initiated from the cleavage of a β-carotene to zeaxanthin. The conversion of zeaxanthin to xanthoxin is carried out in plastids by 9-cis-epoxycarotenoid dioxygenase (NCED). The process takes place in the cytoplasm, where a short-chain alcohol dehydrogenase converts xanthoxin into abscisic aldehyde, which is eventually oxidized to ABA [213,214,215].

The general impact of ABA during ripening is the upregulation of ethylene synthesis genes [216,217]. Also, ABA antagonizes several GA effects, promoting seedling growth and α-amylase synthesis [218]. Meanwhile, abscisic acid is considered an antagonist of brassinosteroids during fruit ripening. The abscisic acid signaling pathway consists of the family of receptor proteins PYR (PYRABACTIN RESISTANCE), PYL (PYR1-like), RCAR (regulatory components of ABA receptors), protein phosphatases PP2C, and SnRK2 kinases. There is evidence of-positive regulation of abscisic acid biosynthesis by BS signaling. BZR1 (brassinazole-resistant) was found to mediate brassinosteroid signaling by promoting abscisic acid biosynthesis through direct transcriptional regulation of *NCED1* [219]. Here, BIN2 negatively regulated BZR1 protein accumulation and cold tolerance by suppressing abscisic acid biosynthesis.

The suppression of *PP2C3* in tomatoes accelerated the onset of fruit ripening and affected their glossiness by changing the external structure of the epidermis [220]. In transgenic plants, an increase in the expression of *SnRK2*, *PYL* receptors, various cutin synthesis and transfer genes, and *CYP* (cytochrome P) genes was observed. The role of PP2C as a negative regulator in abscisic acid signaling was further supported in [221], where the alteration of *PP2C5* expression affected fruit quality traits, including pericarp thickness and shape, seed number, and soluble solid content. In addition, *PP2C1* silencing increased the accumulation of endogenous abscisic acid and accelerated ethylene release in transgenic tomatoes compared to wild-type fruit [222]. *PP2C1*-RNAi lines had abnormal flowers, and pedicel abscission was impaired.

Abscisic acid homeostasis is regulated by its conjugation with glucose using uridine diphosphate glucosyltransferases (UGT). It was shown that RNAi silencing of the *UGT75C1* gene significantly increases the level of expression of the CYP707A2 hydrolase gene while not affecting the expression of the key gene for abscisic acid biosynthesis NCED1 [223]. Suppression of *UGT75C1* significantly accelerated fruit ripening by increasing abscisic acid levels and promoting early ethylene release.

The PYL9 protein has been identified as a positive regulator of abscisic acid signaling [224]. Depending on abscisic acid concentration, PYL9 can inhibit the protein phosphatase PP2C. In tomatoes overexpressing *PYL9*, fruit ripening was significantly accelerated due to the early release of ethylene. The abscisic acid-induced oxidase gene *DAO2* (dioxygenase for auxin oxidation) inhibited hypocotyl elongation in tomatoes, exhibiting an antagonistic role to auxins [225]. SnRK phosphorylation is mediated by the protein kinase MAPK11, thereby regulating abscisic acid biosynthesis and signaling [226].

In addition, a new transcriptional repressor of abscisic acid biosynthesis, EAD1 (ERF-associated amphiphilic repression (EAR) motif-containing ABA downregulated), was recently discovered [227]. Although the authors have not studied the molecular mechanism of repression, its implementation is possible either through the recruitment of histone deacetylases with subsequent formation of a complex with co-suppressors or through direct or indirect binding to transcription factors.

### 4.7. Salicylic Acid Regulation

Salicylic acid (SA) is a phenolic signaling compound coordinating plant responses to pathogens and many physiological and developmental aspects of plant life [228]. SA is synthesized via two distinct pathways in plants: the phenylalanine ammonia-lyase (PAL) pathway and the isochorismate synthase (ICS) pathway [229]. During tomato fruit ripening, there is an increase in the expression of *PAL* but not *ICS* [230]. Endogenous SA regulates ethylene accumulation significantly at later stages of fruit ripening [230]. In this case, negative regulation is observed between an increase in SA concentration and the activity of ethylene synthesis genes [231].

The SA-mediated regulation of tomato fruit ripening appears to be maintained by SA-dependent bZIP transcription factors, namely TGA2 [232]. It has been shown that TGA2-mediated repression alters early fruit development and metabolism, including chloroplast number and structure, considerably slowing fruit ripening. Another transcription factor induced by SA is HDZ28-like, which belongs to the *HD-ZIP* gene family [233]. HDZ28 positively regulates *EDS1* (ENHANCED DISEASE SUSCEPTIBILITY 1), which lies upstream of SA biosynthesis and is essential for activating SA signaling. NAC transcription factor NAP1 activated the transcription of multiple genes involved in both SA and ABA biosynthesis [234]. Evidence shows that SA regulation involves lncRNAs [235]. Also, it appears that the expression of chromatin-remodeling complexes (CHRs) is repressed by SA but enhanced by ABA [236], which gives a clue of the SA-mediated regulation of other hormonal regulatory pathways.

Cis-elements in the promoter region of the wall-associated kinase (WAK) gene, which is a subfamily of receptor-like kinases associated with the cell wall, are susceptible to methyl jasmonate, abscisic acid, and SA [237]. The regulation of ripening genes may involve calcium-dependent protein kinases under the dependency of ethylene and SA [238]. Indeed, expression profiles of calcium-dependent proteins were dramatically altered in ripening mutant *rin* compared with WT [239]. Calcium-dependent proteins have distinct roles in responses to the specific stress signals [240], and they connect calcium-mediated signaling with SA stress signal transduction during fruit ripening and storage [241]. The peroxidase gene, *Prx09*, is found to be expressed in the mesocarp of tomato fruits and was mainly induced by SA and JA. *Prx09* overexpression displayed high resistance to H_2_O_2_ stress [242]. Therefore, SA enhances the anti-oxidative capacity that results in the prolonged shelf life of tomato fruits.

The SA level of tomato fruits is maintained by salicylic acid carboxyl methyltransferase (SAMT), which catalyzes the reaction of SA and the methyl donor S-adenosyl-l-methionine (SAM) to methyl salicylate [243]. Exogenous treatment of tomato fruit with methyl salicylate shows increased ethylene production, and it is possibly mediated by depressing the negative feedback regulation of the *ACS6* genes and increasing the expression of ACS2 and ACS4 through positive feedback regulation [244]. On the contrary, MES (SALICYLIC ACID METHYL ESTERASE) carries out the demethylation of methyl salicylate. Expression of *MES1* and *MES3* is specified only in ripening fruits [245]. Therefore, silencing of *SAMT* or overexpression of methyl esterases in tomatoes can improve the taste of fruits by reducing the concentration of methyl salicylate, which makes fruits bitter, and increase shelf life by increasing the concentration of SA. Additionally, fruit SA’s storage is maintained through 2,5-dihydroxybenzoic acid sugar conjugates [246]. DOWNY MILDEW RESISTANCE 6 (DMR6) catalyzes the hydroxylation of SA [247] and appears to be specialized in balancing SA levels in flowers/fruits [248]. The decarboxylative hydroxylation of SA to catechol is an additional SA degradation reaction in tomatoes catalyzed by FAD/NADH-dependent SA 1-hydroxylase [249].

SA–auxin pathways crosstalk becomes revealed. SA altered the auxin transporter PIN’s polar membrane localization by directly binding to phosphatase PP2A [250]. Auxin response factors were reported to be expressed against SA, and it appears ARF2 downregulates abscisates and SA biosynthesis genes while it upregulates the cytokinins biosynthesis genes [251].

### 4.8. Jasmonate Regulation

Jasmonic acid (JA) is a fatty acid-derived signaling molecule that regulates defense responses against pathogens [252,253,254,255,256,257,258] and abiotic stresses [259,260,261]. Their synthesis from linolenic acid occurs via the octadecanoid pathway [262]. Unfortunately, same as for SA, its roles in ripening have not been extensively studied. SA and JA act antagonistically in resistance to specific pathogen types. SA accumulation represses auxin and JA synthesis by inhibiting catalase activity [263]. Mediator complex MED17 is shown to integrate JA and auxin signaling pathways [264]. BR antagonistically acts upstream of the JA signaling pathway [265].

JA negatively regulates GRFs (GROWTH REGULATING FACTORS), which are positive regulators of GA biosynthesis [266]. Meanwhile, DELLA is shown to repress JA ZIM-domain (JAZ) proteins [267]. Methyl JA is found to promote ethylene production [268]. In Arabidopsis, JA enhances the transcriptional activity of *EIN3/EIL1* by removal of JA-ZIM domain (JAZ) proteins, which repress *EIN3/EIL1* by recruiting histone deacetylase (HDA6) as a corepressor [269].

Jasmonoyl-isoleucine accumulates at the immature fruit stage and then decreases as the fruit ripens [270]. bHLH transcription factor MYELOCYTOMATOSIS 2 (MYC2) is repressed by JAZ [271]. JAZs are targets of the E3 ubiquitin ligase [272]. JAZs and E3 ubiquitin ligase form a jasmonoyl-isoleucine receptor [273] and perform JAZ degradation, releasing MYC2 from repression. MYC2 interacts with the mediator complex MED25 and recruits histone acetyltransferase (HAC1) [274], which epigenetically regulates the transcription of JA-responsive genes. Also, JAZ forms a corepressor complex with NOVEL INTERACTOR OF JAZ (NINJA) and TOPLESS (TPL) [275]. MYC2 is found to regulate growth and fruit quality in tomatoes [276]. MYS2 shows an autoregulatory negative feedback loop in the termination of JA signaling by activation of a group of JA-inducible bHLH proteins, MYC2-TARGETED BHLHs (MTBs), that impair the formation of the MYC2-MED25 complex [277].

It appears that JA acts downstream of ABA. High levels of ABA-induced several ripening-related genes through JA, but not all the ripening-related genes responded to JA [278]. Moreover, an antagonistic relationship from the JA to the ABA pathway during fruit ripening has been proposed [279]. Lipoxygenase (LOX), namely LOX-B, is found to mediate methyl JA accumulation in tomato fruits [280]. Here, the authors stated that methyl JA alters the aminome of ripening fruits. The feedback regulation of *LOX* in response to methyl JA has been recently discussed [281]. *LOX* promoter regions contain cis-acting regulatory elements required to properly regulate *LOX* expression during development and for responsiveness to methyl JA [282]. A MADS-box transcription factor MYB117 seems to upregulate *LOX* and downregulate the methyl JA pathway [283].

Structural cell wall proteins extensins (EXT) have cis-acting elements in the promoter region that are involved in responses to different signal molecules, including JA. Thus, the latest could participate in their regulation [284]. There is evidence that JA regulates the biosynthesis of secondary metabolites through tomato fruit ripening. Upregulation of JA alters the carotenoid biosynthesis metabolite content in ripening tomato fruit [285]. Methyl jasmonate affects the accumulation of caffeoylputrescine [286] and lycopene [268]. JA has been shown to be involved in the expression of genes related to fruit cell wall and anthocyanin metabolism [278]. Additionally, methyl JA is involved in synthesizing volatile organic compounds [287].

### 4.9. Hydrogen Sulfide

Hydrogen sulfide counteracts the effects of ethylene during ripening. The assimilation of sulfates in chloroplasts can produce endogenous hydrogen sulfide, and the main enzymes in this process are sulfite reductases [288]. Cytosolic hydrogen sulfide can also be generated from cysteine by cysteine desulfhydrase 1 (DES1/LCD1). Loss-of-function mutations of *LCD1* in tomatoes [289] increase the expression of genes for ethylene synthesis (*ACO1*, *ACO3*, and *ACS2*), carotenoids (*PSY1*, *PDS*, and *ZDS*), and cell wall metabolism (*CEL2*, *EXP*, *XTH5*, *PG*, and *TBG4*). Knockout of the tomato D-cysteine desulfhydrase (DCD) gene results in increased expression of ripening-related genes, including *NYC1*, *PAO*, *SGR1*, *PDS*, *PSY1*, *ACO1*, *ACS2*, *E4*, *CEL2*, and *EXP* [290].

An attempt to understand the hydrogen sulfide-mediated regulation of ripening is made in [291]. The authors suggest that the ubiquitin–protein ligase BRG3 undergoes persulfidation at two cysteine residues, leading to a decrease in ubiquitinating activity and its interaction with the repressor transcription factor WRKY71. This leads to increased binding of WRKY71 to the promoter of cyanoalanine synthase (CAS1) gene, which inhibits its transcription and, thus, prolongs fruit ripening.

There is also confirmation that hydrogen sulfide is a regulator of aging, which is noticeable in changes in the expression of chlorophyll degradation genes (*NYC1*, *PAO*, *PPH*, *SGR1*) and the aging-associated gene *SAG* [292].

## 5. Abiotic Ripening Factors

Fruit ripening is also regulated by signaling systems activated in response to abiotic stimuli, and light is one of them. In plants, light has two purposes: first, it provides energy for photosynthesis; second, it is an environmental signal that affects a variety of biological processes, including photomorphogenesis, germination, phototropism, and circadian rhythm entrainment [293,294]. It has been reported that changes in light sensitivity and light-sensitive signaling in tomatoes can significantly change fruit development and quality characteristics [295,296]. In this context, phytochromes act as molecular switches in response to light. Phytochromes are photoreceptors to the red and far-red light spectrum [297]. Light exposure promotes the conformation change in phytochromes to an active form. In the cytosol, they regulate the translation of mRNA [298], while in the nucleus, they modulate the transcription of downstream genes [299]. Following light activation, phytochromes deactivate photomorphogenic response repressor proteins (e.g., COP1, CUL4, DDB1, DET1, and PIF).

Using RNAi silencing of the phytochrome genes *PHYA*, *PHYB1*, and *PHYB2*, it was shown that PHYA positively affects the differentiation and division of tomato plastids through changes in the expression of both light-dependent genes and cytokinin-dependent genes [300]. Regulators of carotenoid biosynthesis (GGPS, PSY1, and PDS) were also affected, resulting in decreased carotenoid biosynthesis during fruit ripening.

As for the repressor proteins mentioned above, their effect on ripening is also being studied. Thus, according to [301], overexpression of *COP1* (CONSTITUTIVE PHOTOMORPHOGENIC) from *Solanum melongena* in tomatoes caused a delay in fruit ripening by 3–6 weeks. These transgenic plants showed decreased ethylene production due to suppressing the expression of the central genes of its biosynthesis *ACO1*, *ACO3*, and *ACS2*. The carotenoid biosynthesis genes *PSY1*, *PDS*, and *ZDS* were also downregulated. In [302], using the *DDB1*, *DET1*, and *CYC-B* genes as an example, the multiplex Target-AID (activation-induced cytidine deaminase) technique was developed. As a result, the authors obtained two lines of triple mutants, in which each gene had two-point substitutions, which showed a higher accumulation of carotenoids and lycopene compared to the wild type. In tomatoes, PIF-dependent light signaling has been reported to regulate fruit development and influence nutritional value and ripening time. Transient overexpression of *PIF3* in tomato fruit resulted in decreased *GGDR* mRNA levels, which was inversely related to *PIF3* transcript levels [303]. These data indicate that PIF3 mediates PHY-dependent regulation of tocopherol biosynthesis through transcriptional inhibition of geranylgeranyl diphosphate reductase expression in tomato fruit. Evidence shows that PIF4 can regulate hypocotyl elongation, plant growth, flowering, and leaf senescence in response to light and temperature [304]. The authors support this statement by obtaining tomatoes with RNAi-mediated knockdown of *PIF4*, which showed increased carotenoid content, accelerated fruit ripening time, and delayed leaf senescence. A small number of flowers and a decrease in vegetative mass were observed in such plants. Knockout of *PIF3* using CRISPR/Cas9 led to the arrest of phase I of pollen mitosis, which was reflected in its non-viability [305]. Glutamate synthase (GLT1) and cell wall invertase (CWIN9), involved in auxin and sugar homeostasis, respectively, have also been shown to colocalize with PIF3 in anthers and are directly regulated by PIF3. Knockout lines of *GLT1* and *CWIN9* (cell wall invertase) showed a similar phenotype. VIGS-mediated silencing of the light-signaling transcription factors HY5 and PIF3 led to changes in glycoalkaloid levels in tomato leaves compared to wild type, suggesting their involvement in the regulation of target genes of glycoalkaloid metabolism [306].

While the most abundant antioxidant in tomato fruit is the lipophilic carotenoid lycopene, levels of water-soluble flavonoids (including anthocyanins) are suboptimal. Plants accumulate anthocyanins in response to various stress events such as low temperature, drought, UV radiation, intense light, and nutrient deficiency, acting as an antioxidant and photoprotective agent. The bZip transcription factor HY5 is believed to be a significant regulator of anthocyanin accumulation in plants in response to light [307,308]. However, research [309] has cast doubt on the accuracy of this statement. By creating *HY5*-knockout mutants, the authors demonstrated a reduced anthocyanin content, which suggests the presence of additional pathways for their synthesis independent of HY5. Indeed, eight candidate anthocyanin transcription factors have been identified.

A recent study has uncovered the function of the little-studied PHY-F. It turned out that PHY-F is a low-flux radiation sensor [310]. It forms dimers with PHYA and/or PHYB, with which it makes additive contributions to various processes of photomorphogenesis.

In addition to the phytochromes of red and far-red light receptors, there are also cryptochromes of blue light receptors—CRY1 and CRY2. Tomato lines overexpressing *CRY1a* showed significant accumulation of anthocyanins through the regulation of genes encoding key enzymes of anthocyanin biosynthesis (e.g., *AN2* and *DFR* (dihydroflavonol 4-reductase)) [34]. The same study showed that blue light consistently induced overexpressing tagged HY5 protein accumulation in tomatoes. In addition, it was shown that under the influence of blue radiation, repression of *COP1* (CONSTITUTIVE PHOTOMORPHOGENIC) transcription was observed, which was confirmed by the creation of lines with RNAi-*COP1*. Ultimately, the silencing of *HY5* and two anthocyanin biosynthesis genes (*CHS1* (chalcone synthase) and *DFR*) in *CRY1a* lines was accompanied by a decrease in anthocyanin accumulation. Moreover, CRY1a was found to be critical for regulating starch accumulation in chloroplasts by inducing starch degradation through the transcription factor HY5 [311]. Induction of transcription of genes associated with starch degradation under the influence of blue radiation in *CRY1a*- or *HY5*-overexpressing plants was also confirmed.

It is known that in tomato, the R2R3-MYB group of factors regulating anthocyanin biosynthesis is represented by *AN* (*ANANTHA*) genes. Currently, their biological function is being actively clarified. For example, by generating loss-of-function mutants of AN2, the authors identified it as a positive regulator of anthocyanin biosynthesis in tomato vegetative tissues [312]. In addition to reduced anthocyanin content, the mutants had a dwarf phenotype. Overexpression of *AN2* resulted in changes in multiple fruit qualities [313]. Thus, increased production of ethylene and increased content of anthocyanins, phenols, and flavonoids were observed. The content of aromatic volatiles such as aldehydes, phenylpropanoid derivatives, and terpene volatiles was also increased in these fruits. Thus, AN2 was shown here to regulate the transcription of genes in several metabolic pathways. Additionally, it was found that loss-of-function mutations in the *AN2* ortholog in wild tomato impair anthocyanin synthesis [314]. Overexpression of *ANT1* in tomatoes enriched the anthocyanins in leaves, contributing to more intense light absorption in the blue and red spectrum [315]. However, introducing knockout mutations into the *AN2*-like gene rather than *ANT1* (*ANTHOCYANIN*) essentially eliminates the accumulation of anthocyanins [316,317,318]. It was found that AN2-like activated the expression of *DFR*; however, when *AN1* was knocked out, anthocyanin pigmentation in the fruits was also eliminated. The AN2-like antagonist is the R3-MYB protein MYBATV. Meanwhile, a similar conclusion regarding MYBATV was made earlier [319]. It can be summarized that HY5 activates AN2-like, promotes the expression of *AN1* and *MYBATV*, and MYBATV protein competes with AN2-like for binding to AN1 and thereby negatively regulates anthocyanin biosynthesis. Moreover, in [253], overexpression of AN2-like was found to increase jasmonic acid accumulation, activate the defense signaling pathway against *Botrytis cinerea*, and also increase fruit shelf life by inhibiting the expression of genes associated with the modification cell wall.

The previously mentioned dihydroflavonol 4-reductase (DFR) is involved in the reduction in dihydroflavonols to leukoanthocyanidins during the synthesis of the pigments pelargonidin, cyanidin, and delphinidin. The *DFR* gene in the tomato genome is represented by a single copy, which prompted its use in developing a natural genome editing marker based on homologous recombination with restoration of the DFR function [320]. *DFR* expression is also regulated by BBX20, which binds to its promoter region to activate expression [321].

## 6. System of Regulation of Tomato Fruit Ripening Process

**Figure 4:**
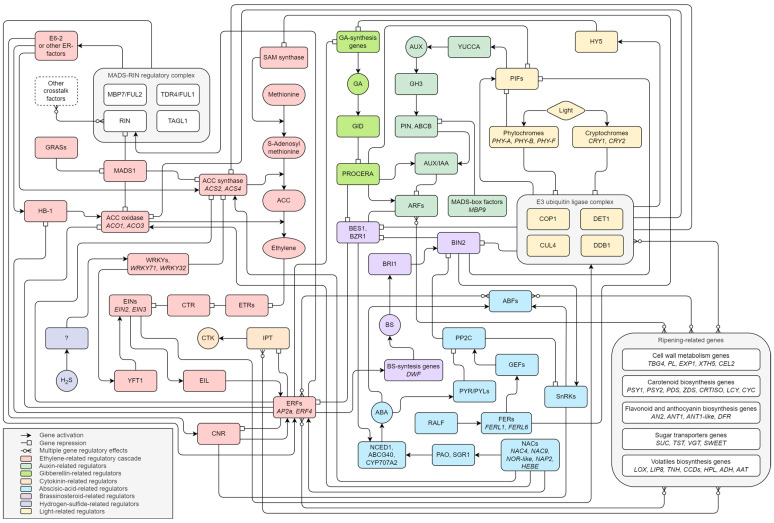
A molecular interaction network model of ripening-related genes in tomato. All interactions are based on experimental data reported in scientific publications. A molecular interaction network model was created using the free online web application draw.io (https://www.drawio.com/ (accessed on 5 January 2024)).

As a result of our literature review, we present a putative model of ripening factor regulatory pathways (Figure 4). We recognize five components of the ripening regulation system: transcription factors, hormones, epigenetics, external stimuli, and ncRNAs.

A significant contribution to regulation is provided by the ethylene-dependent pathway involving important polycistronic regulators like ethylene-sensitive genes, ethylene response genes, and the MADS-RIN complex. The fact that there are many additional factors with which RIN directly interacts suggests the existence of various ad hoc regulatory complexes consisting of several units of transcription factors. Quite often, functional redundancy is observed for them. There are three possible relationships of transcription factors: redundancy, additivity, and dependency. Redundancy is manifested in the functional identity of transcription factors. Additivity is associated with the provision of function through the joint contribution of each element. Direct dependence involves activation or repression of the role of one factor only after interaction with another. Moreover, autocatalytic regulation of the participants of regulatory cascades is possible.

In practice, disruption of the function of ripening-related factors does not interrupt the entire cascade of gene regulation but only leads to a delayed ripening phenotype. Indeed, we show other regulators of the ethylene pathway, including genes from auxin, gibberellin, brassinosteroid, and abscisic acid pathways. They primarily act as negative regulators of ethylene accumulation, mainly the auxins. Although SA and JA pathways are absent in the proposed scheme, we do not exclude the presence of SA and JA regulators as additional ripening factors. We need more studies to clarify their role in these processes to conclude that they contribute significantly to regulating ripening-related genes. Nevertheless, it was evident that SA antagonizes ethylene during fruit ripening and prevents ethylene burst to keep the process of fruit development. Also, SA and JA prevent fruit senescence by reducing ethylene concentration in the late ripening stage.

Activation or deactivation of genes involved in ripening regulation can be mediated epigenetically, as discussed previously with specific examples. RIN-mediated regulation also requires interaction with promoters of lncRNAs, which are regulators of other genes, including those associated with ripening. This provides the so-called ethylene-independent regulatory pathway of ripening genes. Because epigenetic regulation and lncRNA regulation are potentially applicable to each element of regulatory cascades, their display on the scheme is redundant.

## 7. Future Prospects and Challenges

Significant progress has recently been made in understanding signal transduction systems and processes. The discovery of gene function and their regulatory systems in ripening processes allows breeding of tomatoes with an increased amount of fruits and improved nutritional properties.

Although there have been attempts to generalize the crosstalk of hormonal signaling pathway cascades [322,323,324], they are only partially consistent. They share a common concept of the participation of ethylene-dependent genes in tomato fruit ripening. Genes of transcription factors ensure the regulation of these processes. Of course, the proposed concepts still need to be completed. Recently, a conceptual shift in the theory of master regulators of ripening to the redundancy of factors-mediated ripening has been made [325]. There appear to be no master regulators controlling the ripening process but a group of redundantly acting homologous genes. They can be studied by assessing the effect of combined mutations, which are now available by multiplexed CRISPR/Cas9 mutagenesis. The molecular triggers of instantaneous ethylene burst in fruits are yet clear. Moreover, new findings in the participation of epigenetic modification [38,51,54,95,326,327,328,329] and ncRNA [9,330,331,332,333] in the regulatory process provide new grounds for revising established molecular interactions of signal complexes [334]. Available studies indicate that the regulatory elements that affect tomato fruit ripening work in concert rather than alone. There is not yet enough depth of knowledge of these cooperative processes. Future studies must investigate the interactions among histone modification, ncRNA, and NA-methylation modifications to gain a complete regulatory network for tomato fruit ripening. Although the abiotic triggers of tomato flower development, fruit set, and development are pretty abundant, there is a lack of knowledge about the abiotic-mediated regulation of ripening. Therefore, this area should be enriched, too. All of this can be achieved with novel biotechnological tools.

All this reminds us of a puzzle: to start putting it together, you need to find the edge of the image; otherwise, it can take infinite time to compare individual elements. But signaling pathways do not have “edges”; they must be created manually. One of the modern approaches to solving problems in this area is the use of a wide range of genetic engineering and bioengineering methods, including, among other things, the collection and processing of bioinformatics data, new sequencing technologies, targeted genome editing using CRISPR technology [335,336,337,338,339,340,341,342], the use of base and prime editing for precision gene correction [343,344], the creation of unique and simple markers for detection of transgenes [345], application transcriptomics, metabolomics and other omics technologies.

However, even here, some difficulties arise. For example, orthologous genes often have different regulatory mechanisms and are unreliable predictors of expression in related species. In addition, not all studies are field-validated, which reduces the significance of the data obtained using transgenic plants. Ideal and, at the same time, simplified laboratory conditions cannot determine all the possible subtleties of gene expression and their regulation. Moreover, changes in the expression of regulatory genes do not always entail significant changes in the transcriptome due to the possible presence in the genome of paralogs of the genes being studied. For genes higher in the regulatory cascades, pleiotropic changes occur. It is worth mentioning that the reproduction processes are discrete and appear in various tissues with excellent synchronization, determined by the life cycle stage, so their identification and description are difficult.

Undoubtedly, the existing findings about the regulation of plant life cycle processes obtained on model objects such as the tomato, although extensive, still need to be completed. In the future, genetic approaches will continue to make essential contributions to identifying new candidate genes involved in tomato reproductive signaling cascades. This may open up a broader cluster of regulatory signaling networks involving currently unknown factors and stimuli.

As for the perspectives of the practical application of the study of the considered ripening-related genes, these are improving fruit quality due to increased nutrient content, accelerated ripening, prolonged shelf life, and much more. However, improving certain traits is usually possible by transferring expression cassettes into the plant genome. Such plants with an altered genome can be considered genetically modified (GM). GM organisms are widely used for various purposes in fundamental and applied research. Despite this, GM crops still cause negative perceptions among society due to their potential human health problems and horizontal gene flow to non-target organisms [346,347]. Consequently, transnational corporations are exploring and developing modern biotechnological methods for crop improvement. These include rejecting marker, viral, and bacterial genes; creating cis- and intragenic plants; precise gene editing; and others [348]. The pace of regulation in many jurisdictions has not kept up with scientific progress; old paradigms and regulatory frameworks for conventional GMOs must be reevaluated to accommodate new developments. This is possible through international coordination among all stakeholders, including scientists, policy makers, farmers, and members of the public.

## 8. Conclusions

This review examined recent advances in studying tomato ripening factors using various gene engineering approaches. The abundance of current scientific reports cited in this review article reflects the convenience of tomato as a model crop and the breadth of approaches and methods. Despite significant advances, an abundance of biochemical pathways, the involvement of hundreds of genes in the fruit ripening process, and fine regulation involving transcription factors and ncRNAs, it is too early to talk about a complete understanding of the described processes.

The presented research into the factors of tomato fruit ripening continues to expand our understanding of the molecular and physiological basis of these processes, which has significant applications for improving breeding methods and growing new varieties with enhanced phenotypic traits that meet the requirements of the modern agricultural industry and consumer demand. The proposed model of gene regulation will allow us to understand the mechanism of tomato fruit ripening better and complement the overall knowledge picture.

## Data Availability

Not applicable.

## Supplementary Materials

The following supporting information can be downloaded at: https://www.mdpi.com/article/10.3390/ijms25020760/s1.
