# Supplementary material for: Recent Advances in Studying the Regulation of Fruit Ripening in Tomato Using Genetic Engineering Approaches
Source: Int J Mol Sci. 2024 Jan 7;25(2):760. doi: 10.3390/ijms25020760 (PMC10815249; doi:10.3390/ijms25020760)
Supplement: Supplementary file 1 [file ijms-25-00760-s001.zip › ijms-2778023-supplementary.pdf]

Supplementary Figures

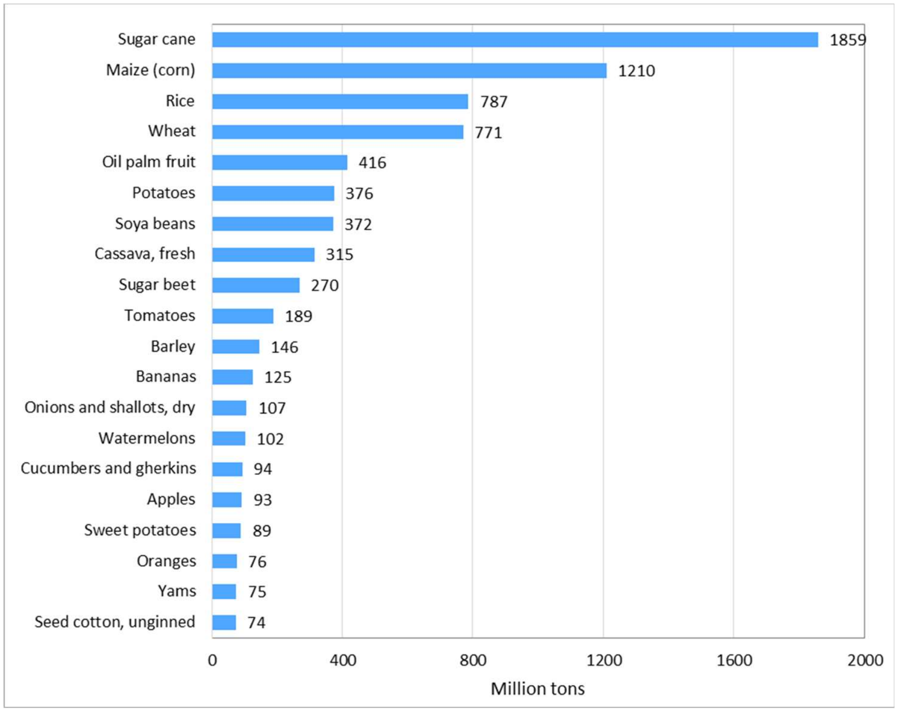

Figure S1. Worldwide crop production: Top 20 in 2021. Source: FAOSTAT (https://www.fao.org).

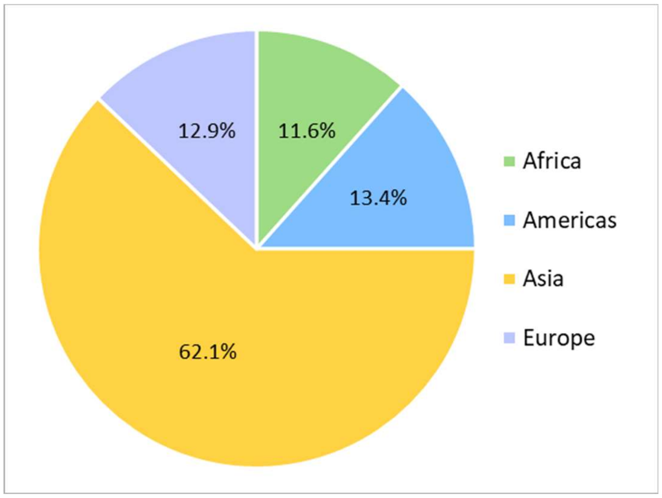

Figure S2. Production share of tomatoes by region. Average values from 2017 to 2021 are shown. Source: FAOSTAT (https://www.fao.org).

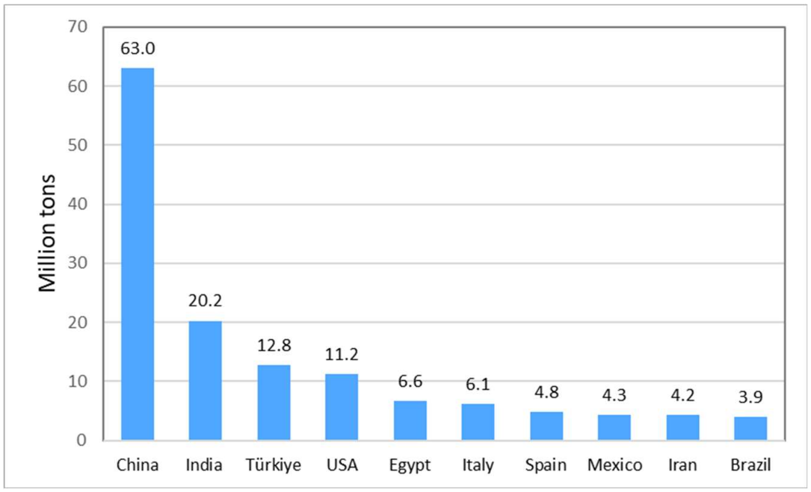

Figure S3. World crop production by country (top 20 leaders are shown). Source: FAOSTAT (https://www.fao.org).

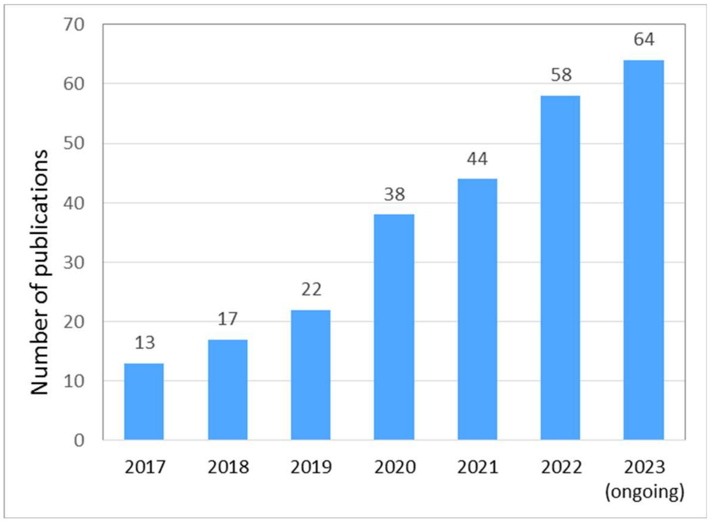

Figure S4. CRISPR/Cas9-related studies on tomato by year.
